# Parasitics-Aware Quantum Transport Simulation of Stacked Si Nanosheet LGAA-nFETs for Sub-2 nm Node RF Applications

**DOI:** 10.3390/mi17020240

**Published:** 2026-02-12

**Authors:** Qi Shen, Shuo Zhang, Zhi-Fa Zhang, Wenchao Chen, Zekai Zhou, Sichao Du, Hao Xie, Wen-Yan Yin

**Affiliations:** 1 College of Integrated Circuits, Zhejiang University, Hangzhou 311200, China; 22547117@zju.edu.cn; 2 College of Information Science and Electronic Engineering, Zhejiang University, Hangzhou 310027, China; 22431017@zju.edu.cn (Z.-F.Z.); 22531158@zju.edu.cn (Z.Z.); sichaodu@zju.edu.cn (S.D.); xieh@zju.edu.cn (H.X.); 3 ZJU-Hangzhou Global Scientific and Technological Innovation Center, Hangzhou 311200, China; 4 School of Integrated Circuits, Shanghai Jiao Tong University, Shanghai 200240, China; wenchaochen@zju.edu.cn; 5 School of Information and Electrical Engineering, Hangzhou City University, Hangzhou 310015, China

**Keywords:** crystal orientation, dual-*k* spacer, non-equilibrium Green’s function (NEGF), operational frequency, parasitic effect, two-band *k·p*, stacked nanosheet transistors, terahertz application

## Abstract

This work presents a comprehensive quantum transport modeling and simulation framework to evaluate parasitic effects and radio frequency (RF) performance in stacked silicon (Si) nanosheet (NS) lateral gate-all-around (LGAA) nFETs targeting the sub-2 nm technology node. Leveraging the non-equilibrium Green’s function (NEGF) method, the proposed framework integrates detailed modeling of parasitic resistances (*R*_para_) and capacitances (*C*_para_) to enable a holistic analysis of both intrinsic and extrinsic figures-of-merit, including transconductance (*g*_m_), output conductance (*g*_d_), cutoff frequency (*f*_T_), and maximum oscillation frequency (*f*_max_). The effects of nanosheet geometry, crystal orientations, and dual-*k* spacers on high-frequency performance are systematically investigated. The analysis reveals key design trade-offs, with optimized device configurations yielding *f*_T_ exceeding 400 GHz and *f*_max_ approaching 1.2 THz. These findings highlight the potential of stacked NS LGAA-nFETs for future millimeter-wave and terahertz applications, providing critical insights into parasitics management and quantum-transport-aware design strategies at advanced CMOS nodes.

## 1. Introduction

The relentless advancement of wireless communication systems, the Internet of Things (IoT), medical imaging, military and aerospace applications has imposed unprecedented demands on the high-frequency performance of CMOS integrated circuits (ICs) [1,2,3]. Particularly, the transition toward 6G and beyond requires carrier frequencies extending into the terahertz range, which imposes even more stringent requirements on the high-frequency figures-of-merit of CMOS ICs [4]. To realize high-efficiency wireless applications and advanced terahertz detection at such frequencies, it is essential to characterize non-linear carrier dynamics and phase transitions using rigorous physical models [5].

As the cornerstone of CMOS technology, transistors must simultaneously deliver high drive current and low parasitic losses to meet the increasingly challenging requirements of advanced technology nodes [6]. While FinFETs have served as the foundation of CMOS ICs for over a decade, their inherent limitations in gate electrostatic control, channel width scalability, and current drivability have prompted the exploration of lateral gate-all-around (LGAA) architectures for next-generation transistors [7,8,9]. In particular, stacked silicon (Si) nanosheet (NS) LGAA-FETs, with their enhanced gate control, higher effective channel width, and improved short-channel immunity, are widely regarded as the most promising successor of FinFET to extend Moore’s Law beyond 2 nm technology node [10,11,12]. On one hand, the aforementioned advantages may help reduce the drain field penetration and output conductance to maintain signal integrity, making the device structure ideal for both logic and analog/RF applications [13,14]. On the other hand, the intricate multi-stack structure can introduce more parasitic capacitances (*C*_para_) and resistances (*R*_para_) that degrade RF performance metrics such as cutoff frequency (*f*_T_) and maximum oscillation frequency (*f*_max_) [15,16]. However, existing studies on stacked Si NS LGAA n-type FETs (nFETs) have predominantly focused on DC performance for logic applications [17,18,19,20]. The channel width, thickness, and crystal orientation configurations of the NS channel have been demonstrated to significantly influence carrier mobility and DC characteristics [21,22,23]. Spacer engineering has been proposed to decouple gate electrostatic control from parasitic coupling [24,25,26]. For example, dual-*k* spacers consisting of low-*k* outer layers and high-*k* inner layers have shown promise in reducing *C*_para_ and OFF-state leakage without compromising short channel immunity [25,26]. Nevertheless, their impact on the RF performance of Si NS LGAA-nFETs has to be analyzed in conjunction with the interdependent effects of channel geometry, crystal orientation, and contact materials. While recent studies, often utilizing commercial TCAD tools such as Synopsys Sentaurus [27,28], have made significant progress in analyzing RF performance, a holistic analysis of silicon nanosheets that integrates these parasitic effects with a more fundamental quantum transport simulation (NEGF) remains underexplored. Addressing parasitic effects together with quantum effects remains a critical challenge for sub-2 nm node RF applications. Prior works explored the RF-oriented design of Si NS LGAA-FETs with the help of commercial solvers like Sentaurus and specialized field solvers like Raphael [29,30,31], but they often oversimplified the quantum transport properties, such as sub-band splitting and non-parabolicity, which are pivotal for sub-2 nm channels. This necessitates the development of a quantum-accurate, parasitics-aware simulation framework that bridges carrier transport with device high-frequency response. While industry-standard compact models, such as the BSIM-CMG, are widely used for circuit-level design, they increasingly rely on quantum-mechanical corrections to account for nanoscale phenomena. High-order simulation frameworks like the NEGF method are critical for benchmarking these compact models, providing the physical basis for their parameter extraction in the absence of experimental data. The framework presented in this work bridges the gap between fundamental quantum transport and technology pathfinding, ensuring that the predictive design rules for sub-2 nm nodes are grounded in rigorous physics.

This article is a revised and expanded version of [32]. Based on the non-equilibrium Green’s function (NEGF) formalism, this work demonstrates a holistic simulation framework that integrates dissipative quantum transport, parasitics modeling, and RF performance evaluation for stacked Si NS LGAA-FETs. Instead of the widely used effective mass approximation model [33,34], a two-band *k·p* Hamiltonian is employed so that the effects of non-parabolicity, sub-band splitting, and quantum confinement on conduction band structure can be captured more explicitly [32,35]. Considering different configurations of channel dimensions, crystal orientation, dual-*k* spacer, and contact materials, parasitic effects and RF figures-of-merit are analyzed systematically for the sub-2 nm technology node. The remainder of this paper is organized as follows: Section 2 details the device structure, parasitic modeling, and the complete simulation framework. Section 3 presents and analyzes numerical results on parasitics and device performance metrics. Section 4 concludes the work.

## 2. Modeling and Simulation Framework

### 2.1. Device Structure Modeling

**Figure 1:**
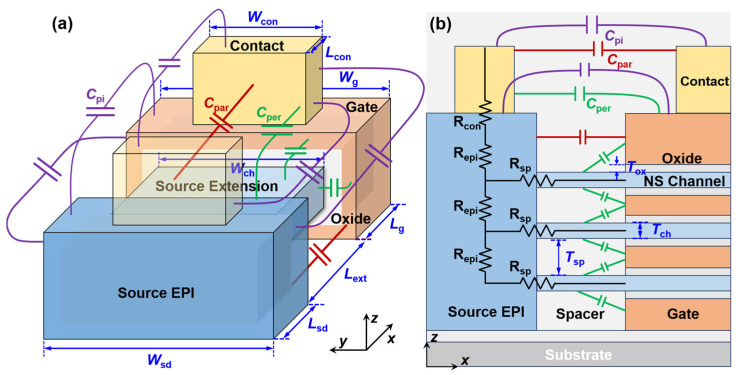
Stacked NS LGAA-nFET structure with various parasitic components in (**a**) three-dimensional schematic of single-stack NS LGAA-FET and (**b**) *x*–*z* cross-sectional view of 3-stack NS LGAA-FETs. Source and drain regions are symmetric with respect to the gate.

Figure 1a and Figure 1b present the three-dimensional (3-D) and cross-sectional schematics of a Si NS LGAA-FET structure, respectively, where all parasitic resistance and capacitance components are explicitly depicted. The *x*–, *y*–, and *z*–coordinate axes align with the device’s length (transport direction), width, and height/thickness, respectively. The metal contacts are positioned atop the source (S)/drain (D) epitaxial (EPI) and the gated regions, with silicide serving as an interfacial layer at the S/D contact. A dual-*k* spacer consisting of Si_3_N_4_ and HfO_2_ is deposited within the gaps between metal contacts, encapsulating the entire extension (EXT) region. The geometric parameters of the device are defined and summarized in Table 1, whose default values are configured to comply with sub-2 nm node specifications according to the 2023 International Roadmap for Devices and Systems (IRDS) [6].

### 2.2. Parasitic Components Modeling

**Figure 2:**
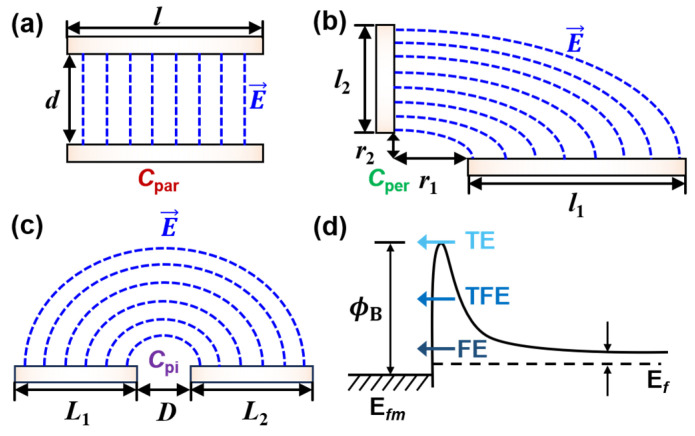
Geometries and electric field distributions of (**a**) parallel-plate, (**b**) perpendicular-plate, and (**c**) co-planar-plate capacitor models. (**d**) Schematics of Schottky barrier formed at the metal contact/Si interface, where the current conduction mechanism contains the competition of thermionic emission (TE), thermionic-field emission (TFE), and field emission (FE).

The total *C*_para_ arises from the electrostatic coupling between various conductive regions of the device, as illustrated in Figure 1, which can be broken down into several key components: the contact-to-contact capacitance, gate-to-contact capacitance, gate-to-EPI capacitance, and gate-to-EXT capacitance. These components can be modeled using parallel-plate (*C*_par_), perpendicular-plate (*C*_per_), and co-planar-plate (*C*_pi_) capacitors models, with their geometric configurations depicted in Figure 2a and Figure 2b, and Figure 2c, respectively.

To clarify the field-coupling topology of the parasitic capacitance network illustrated in Figure 1b, the labeled components are described below from the top of the device to the substrate. The highest red symbol represents the parallel-plate capacitance formed between the inner vertical sidewalls of the two metal contacts. Moving down, the green symbols represent the contact-to-gate and contact-to-S/D EPI fringing perpendicular-plate capacitance. It captures the electrostatic coupling between the vertical sidewall of the metal contact and the top horizontal surfaces of the gate electrode and S/D epitaxy. In the middle region of the structure, the red symbol represents the parallel capacitance formed between the vertical sidewalls of the gate electrode and the S/D EPI. This is a major component of the gate-to-S/D parasitic coupling. At the lower levels near the nanosheet channels, the multiple green symbols represent the perpendicular fringing capacitances between the gate stack and the S/D extension regions across the inner spacers. These *C*_per_ components model the fringing fields emanating from the gate to the extension regions that are not directly parallel. In addition, all the purple symbols represent the coplanar capacitance components between different conductive surfaces, contributed by the coupling of contact-to-contact, contact-to-S/D EPI, and gate-to-S/D EPI regions.

*C*_par_ is calculated as *εlw*/*d*, where *ε* represents the constant and uniform permittivity of the dielectric material between two parallel conductive plates with length *l*, width *w*, and separation distance *d*. Leveraging conformal mapping and elliptic integral techniques, both *C*_per_ and *C*_pi_ can be calculated via an inclined-plate capacitor model [36]. The inner capacitance between two conductive plates inclined at an angle *φ* is formulated as

(1) $$C_{\varphi }(k)=\varepsilon w\frac{K(k)}{K(k^{\prime })}$$

where *φ* is *π*/2 and *π* for *C*_per_ and *C*_pi_, respectively. *K*(*k*) is the complete elliptic integral of the first kind, and the modulus *k* is given by

(2) $$k=\sqrt{\frac{\left({\left(r_{1}+l_{1}\right)}^{\pi /\varphi }-r_{1}^{\pi /\varphi }\right)\left({\left(r_{2}+l_{2}\right)}^{\pi /\varphi }-r_{2}^{\pi /\varphi }\right)}{\left({\left(r_{1}+l_{1}\right)}^{\pi /\phi }+r_{2}^{\pi /\varphi }\right)\left({\left(r_{2}+l_{2}\right)}^{\pi /\varphi }+r_{1}^{\pi /\varphi }\right)}}$$

and $k^{\prime }=(1-k^{2}{)}^{1/2}$.

The minimal distances of the plates to the origin are *r*_1_ and *r*_2_, the lengths of which are *l*_1_ and *l*_2_, respectively. Substituting the corresponding values of *φ*, *r*_1_, *r*_2_, *l*_1_, and *l*_2_ into (2) for *C*_per_ and *C*_pi_ based on Figure 2b,c, we have

(3) $$k_{\mathrm{per}}=\sqrt{\frac{\left({\left(r_{1}+l_{1}\right)}^{2}-r_{1}^{2}\right)\left({\left(r_{2}+l_{2}\right)}^{2}-r_{2}^{2}\right)}{\left({\left(r_{1}+l_{1}\right)}^{2}+r_{2}^{2}\right)\left({\left(r_{2}+l_{2}\right)}^{2}+r_{1}^{2}\right)}}$$

(4) $$k_{\mathrm{pi}}=\sqrt{\frac{L_{1}L_{2}}{\left(L_{1}+D\right)\left(L_{2}+D\right)}}$$

As illustrated in Figure 1, the total *R*_para_ can be divided into contact resistance (*R*_con_), S/D EPI resistance (*R*_epi_), spreading resistance (*R*_sp_), and S/D EXT resistance (*R*_ext_). *R*_para_ is modeled as the *R*_con_ in series with the parallel combination of three identical branches, one for each nanosheet channel. Each of these branches consists of the series combination of the *R*_epi_, *R*_sp_, and *R*_ext_. In our framework, *R*_ext_ can be well handled by the dissipative NEGF simulation; the other extrinsic components are modeled analytically based on established formulas. *R*_epi_ and *R*_sp_ can be calculated by

(5) $$R_{\mathrm{epi}}=\rho_{\mathrm{SD}}\frac{T_{\mathrm{sp}}}{W_{\mathrm{sd}}L_{\mathrm{sd}}}$$

and

(6) $$R_{\mathrm{sp}}=\frac{\rho_{\mathrm{SD}}L_{\mathrm{sd}}}{W_{\mathrm{ch}}T_{\mathrm{sp}}-\left(W_{\mathrm{sd}}-W_{\mathrm{ch}}\right)T_{\mathrm{ch}}}\ln\left(\frac{W_{\mathrm{ch}}\left(T_{\mathrm{ch}}+T_{\mathrm{sp}}\right)}{W_{\mathrm{sd}}T_{\mathrm{ch}}}\right)-\frac{\rho_{\mathrm{SD}}L_{\mathrm{sd}}}{W_{\mathrm{sd}}\left(T_{\mathrm{ch}}+T_{\mathrm{sp}}\right)}$$

where *ρ*_SD_ is the bulk resistivity of n-doped Si. *R*_sp_ results from the mismatch of current pathway at the spreading region from sheet EXT to bulk EPI.

*R*_con_ arising from the current crowding effect and the Schottky barrier (SB) at the contact/Si interface, is calculated via transmission line model (TLM) as

(7) $$R_{\mathrm{con}}=\frac{\rho_{\mathrm{con}}}{\sqrt{\frac{\rho_{\mathrm{con}}}{\rho_{\mathrm{SH}}}}\cdot W_{\mathrm{cnt}}}\cot h\left(\frac{L_{\mathrm{con}}}{\sqrt{\frac{\rho_{\mathrm{con}}}{\rho_{\mathrm{SH}}}}}\right)$$

where *ρ*_SH_ is the n-Si sheet resistivity, and both *ρ*_SD_ and *ρ*_SH_ can be calculated from the electron mobility via the Masetti model [37]. *ρ*_con_ is the contact resistivity, and it is determined by the competition of thermionic emission (TE), field emission (FE), and thermionic-field emission (TFE) at the SB region, as illustrated in Figure 2d. We have

(8) $$\rho_{\mathrm{con},\mathrm{TE}}=\frac{k_{B}}{q_{0}A^{*}T}\exp\left(\frac{\phi_{B}}{k_{B}T}\right)$$

(9) $$\rho_{\mathrm{con},TFE}=\frac{k_{B}}{q_{0}A^{*}T}\sqrt{\frac{E_{00}}{\pi (\phi_{B}+E_{f})}}\cosh\left(\frac{E_{00}}{k_{B}T}\right)\coth\frac{E_{00}}{k_{B}T}\exp\left(\frac{\phi_{B}+E_{f}}{k_{B}T}-\frac{E_{f}}{k_{B}T}\right)$$

(10) $$\rho_{\mathrm{con},\mathrm{FE}}=\frac{\frac{k_{B}}{q_{0}A^{*}}\exp\left(\frac{\phi_{B}}{E_{00}}\right)}{\frac{\pi T}{\sin\left(\pi ck_{B}T\right)}-\frac{1}{ck_{B}\exp\left(cE_{f}\right)}}$$

where

(11) $$E_{00}=\frac{q_{0}h}{4\pi }\sqrt{\frac{N_{\mathrm{SD}}}{\varepsilon_{s}m_{t}^{*}}}$$

(12) $$E_{0}=E_{00}\sqrt{\cot h\left(\frac{E_{00}}{k_{B}T}\right)}$$

(13) $$c=\frac{1}{2E_{00}}\ln\left(\frac{4\phi_{B}}{E_{f}}\right)$$

the effective Richardson constant A* is expressed as

(14) $$A^{*}=\frac{4\pi q_{0}m^{*}k_{B}^{2}}{h^{3}}$$

where *φ_B_* and *E_f_* are the SB height and the Fermi level, respectively.

### 2.3. NEGF-Based Simulation Framework

The comprehensive set of physical parameters, material constants, and simulation targets utilized in our numerical framework is summarized in Table 2.

The detailed execution flow and the hierarchical self-consistent iteration procedures of the in-house dissipative NEGF-Poisson solver are outlined in Table 3.

**Figure 3:**
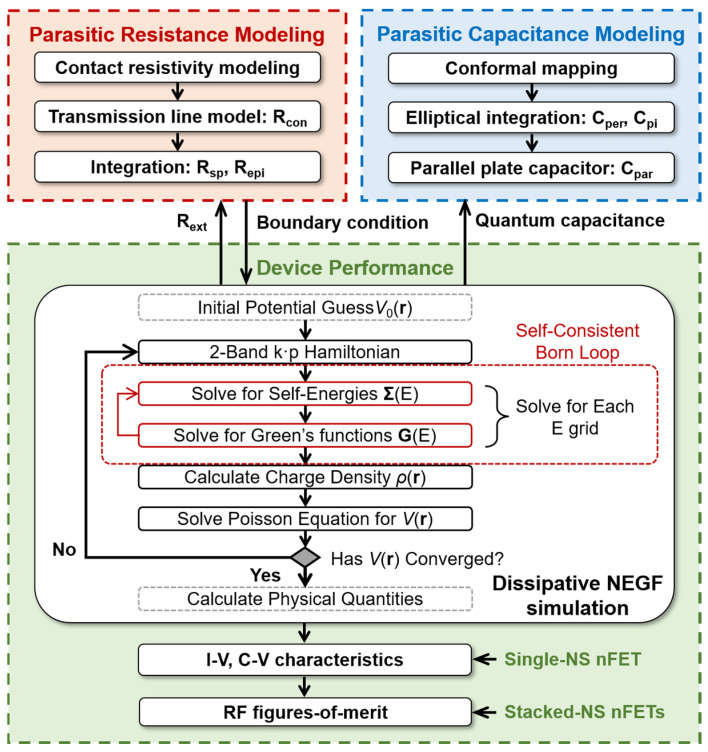
Modeling and simulation framework for evaluating parasitic effects and device performance of the stacked Si NS LGAA-nFETs, where the flowchart of dissipative NEGF simulation is included.

Figure 3 presents our modeling and simulation framework, designed to holistically evaluate the performance of stacked Si NS LGAA-nFETs by linking quantum transport with extrinsic parasitic effects. Our methodology is to first use our in-house developed NEGF simulator to yield the intrinsic device characteristics, which simultaneously accounts for *R*_ext_ and quantum capacitance effects. These intrinsic results are then combined with the analytically modeled extrinsic parasitic components to evaluate the final RF figures-of-merit. The simulation workflow iteratively solves the NEGF and Poisson’s equations through an outer loop for achieving self-consistent distributions of carrier density and electrostatic potential, where the reduced-order mode-space method is adopted to reduce the computational load [38]. Using the unitary transformation matrix constructed by the reduced basis, the two-band *k·p* Hamiltonian, self-energies, and Green’s functions are computed in a reduced-order mode-space for each energy grid. Electron–phonon interactions are incorporated through the self-consistent Born approximation [39], where the retarded and lesser self-energies of acoustic and optical phonon scattering are coupled with Green’s functions in the expressions, requiring an inner iteration loop to realize self-consistency.

As illustrated in the modeling and simulation framework in Figure 3, the tool is structured as a hierarchical evaluation system for advanced technology nodes. The physical core of the framework is the dissipative NEGF engine, which addresses the fundamental transport limits of sub-2 nm nanosheets. This engine utilizes a two-band *k·p* Hamiltonian, which is discretized using the finite difference method across all three spatial directions to accurately capture non-parabolicity and sub-band splitting. To ensure physical rigor while maintaining computational tractability, a reduced-order mode-space technique is implemented via a unitary transformation matrix. Conceptually, the framework bridges pure transport physics and RF engineering through two nested loops: (i) an inner self-consistent Born loop to model carrier energy relaxation via acoustic and optical phonon scattering; and (ii) an outer Poisson loop to achieve electrostatic self-consistency across the 3-D device domain. Furthermore, the framework integrates extrinsic parasitics derived from the TLM for resistances and conformal mapping with elliptical integrals for capacitances. Technically, the calculated parasitic resistances are integrated as voltage-drop elements that self-consistently modify the effective boundary potential of the NEGF solver, ensuring that the finalized RF figures-of-merit (*f_T_*, *f_max_*) incorporate both intrinsic quantum limits and structural parasitic losses.

Furthermore, key RF figures-of-merit, including extrinsic transconductance (*g*_mx_), output conductance (*g*_dx_), *f*_T_, and *f*_max_, are evaluated via

(15) $$g_{mx}=\frac{g_{m}}{1+R_{S}g_{m}},g_{m}={\left.\frac{\partial I_{D}}{\partial V_{GS}}\right|}_{V_{DS}}$$

(16) $$g_{\mathrm{dx}}=\frac{g_{d}}{1+(R_{S}+R_{D})g_{d}},g_{d}={\left.\frac{\partial I_{D}}{\partial V_{\mathrm{DS}}}\right|}_{V_{\mathrm{GS}}}$$

(17) $$f_{T}=\frac{g_{m}}{2\pi C_{\mathrm{GG}}}$$

(18) $$f_{max}=\frac{f_{T}}{2\sqrt{g_{d}(R_{S}+R_{G})+2\pi f_{T}(R_{S}+R_{G})C_{\mathrm{GD}}}}$$

*R_S_* and *R_D_* denote the source and drain resistances, respectively, and they are equal to half of the total resistance under the assumption that they are symmetric. *R_G_* is the gate metal resistance. *C_GG_* is the gate capacitance and can be treated as the sum of parasitic and oxide capacitance [29].

All the simulation parameters are determined according to Table 1 and Table 2 and the device performance specifications in the 2023 IRDS [6]. For high-performance applications at a sub-2 nm node, the OFF-state current (*I_OFF_*) is targeted at 10 nA, the supply voltage (*V_DD_*) is set to 0.6 V, and the operational temperature T is maintained at room temperature (300 K).

## 3. Results and Discussion

### 3.1. Intrinsic Quantum Transport Properties and DC Characteristics

**Figure 4:**
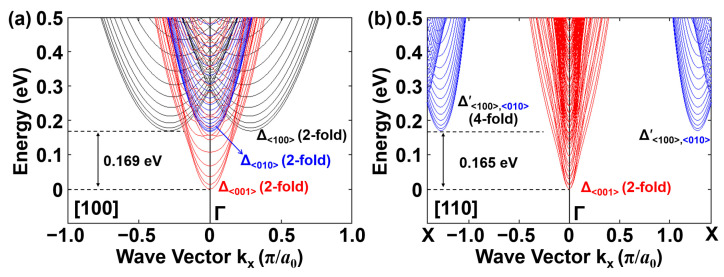
Conduction band structures for Si NS channel with 3 nm × 21 nm cross section oriented along (**a**) [100] and (**b**) [110].

The energy–wave vector (*E*-*k*) dispersion curves of different conduction band sub-bands are shown in Figure 4 for a Si NS channel with 3 nm × 21 nm cross section, obtained from the two-band *k·p* Hamiltonian. Figure 4a,b compares the effects of channel orientations [100] and [110], revealing significant differences in band curvature, degeneracy, and subband splitting. For both orientations, the conduction band minimum resides at the *Γ* point and is projected from the two Δ valleys on the <001> axis, whose sub-bands dominate the electron transport properties. A valley splitting slightly below 0.17 eV is observed in both cases, identifying the energy level of the second lowest valley minimum contributed from the valleys on the <100> and <010> axes. While Δ_<100>_ and Δ_<010>_ valleys are projected onto the [100]-channel with 2-fold degeneracy, the projection onto the [110]-channel merges these valleys near the *X* point, leading to coincident primed sub-bands with 4-fold degeneracy. Moreover, the [110] orientation results in larger density-of-states (DOS) and lower electron effective mass.

**Figure 5:**
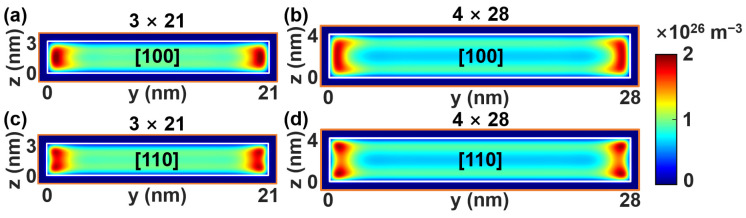
Spatial electron density distributions in the y–z cross-section of ON-state channel (obtained from NEGF simulation) with orientation and cross-sectional area of (**a**) [100]/3 nm × 21 nm, (**b**) [100]/4 nm × 28 nm, (**c**) [110]/3 nm × 21 nm, and (**d**) [110]/4 nm × 28 nm, respectively.

Figure 5 presents the cross-sectional ON-state electron density distributions in the *y*–*z* plane, for NS Si channels with different orientations and dimensions, which are obtained from the dissipative NEGF simulations. For both channel orientations, two prominent conductive layers with high electron concentration emerge adjacent to the vertical sidewalls (aligned with the thickness direction). While the [100]-channel exhibits stronger electron density localization near the midpoint of channel sidewalls, electron density maxima shift toward the four corners in the [110]-channel. This is the consequence of the valley degeneracy phenomenon depicted by Figure 4. Enlarging the cross-sectional area reduces the maximum electron density and enhances spatial uniformity, reflecting mitigated electrostatic confinement. It should be noted that while quantum effects may be addressed phenomenologically in single-device current-voltage (*I*-*V*) characteristics, their dominance is clearly observed in the performance sensitivity to structural scaling. For example, the non-linear shifts in current and capacitance as nanosheet thickness varies are intrinsic manifestations of sub-band splitting and confinement, physics that our NEGF framework captures inherently, offering predictive capabilities that classical drift–diffusion models lack without extensive recalibration.

**Figure 6:**
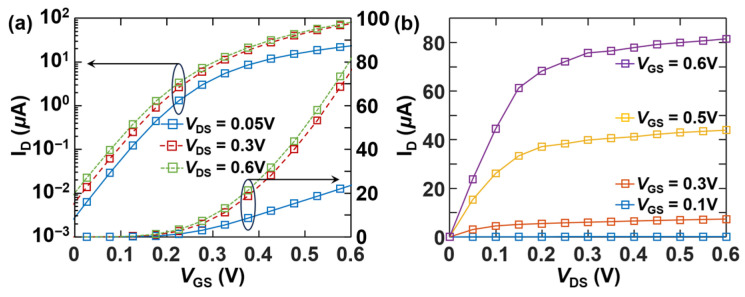
(**a**) *I*_D_-*V*_GS_ transfer characteristics and (**b**) *I*_D_-*V*_DS_ output characteristics at various *V*_DS_ for an LGAA-nFET with 3 nm × 21 nm Si NS channel oriented along [110] direction, where the supply voltage *V*_DD_ = 0.6 V and *I*_OFF_ = 10 nA for the high-performance logic applications.

Figure 6a and Figure 6b present the NEGF simulated transfer and output characteristics, respectively, for a [110]-oriented Si NS LGAA-nFET with a 3 nm × 21 nm sheet cross section. It is observed that I_D_ is saturating as *V*_DS_ approaches *V*_DD_, and an ON–OFF ratio higher than 8 × 10^3^ can be achieved for *V*_DD_ = 0.6 V and *I*_OFF_ = 10 nA. A subthreshold swing (*SS*) of ~76 mV/dec is extracted, indicating effective gate control.

### 3.2. Extrinsic Parasitics Effects and RF Performance

**Figure 7:**
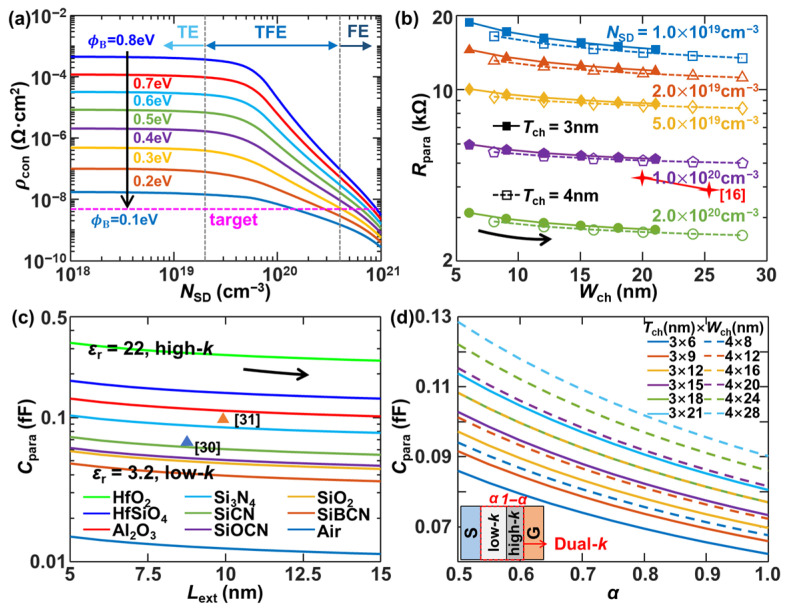
(**a**) Contact resistivity (*ρ_con_*) versus N_SD_ for various barrier heights. (**b**) Source/drain parasitic resistance (*R_para_*) as a function of channel width (*W_ch_*). (**c**) Parasitic capacitance (*C_para_*) as a function of extension length (*L_ext_*) for different spacer materials. (**d**) *C_para_* versus dual-*k* ratio *α*. In (**b**,**c**), the scatter symbols represent independent validation data extracted from recent state-of-the-art studies [16,30,31] for similar nanosheet architectures.

Figure 7a illustrates the dependence of *ρ*_con_ on *N*_SD_ at the S/D contact/n-Si interface for SB height ranging from 0.1 to 0.8 eV. In the low doping regime where *N*_SD_ < 2 × 10^19^ cm^−3^, TE governs the interfacial carrier transport, resulting in relatively high *ρ*_con_ values (>10^−8^ Ω·cm^2^) constrained by the SB height. As *N*_SD_ increases to a moderate level, TFE becomes the dominant conduction mechanism, causing a gradual reduction in *ρ*_con_ with higher doping densities. Within this range, a target *ρ*_con_ of 5 × 10^−9^ Ω·cm^2^ is achievable for SB heights ≤ 0.3 eV. For the degenerate doping case (*N*_SD_ > 4 × 10^20^ cm^−3^), *ρ*_con_ exhibits a sharper decline due to FE-dominated tunneling through the thinned SB. Notably, the effect of SB height diminishes progressively with increasing *N*_SD_. Figure 7b demonstrates that the total *R*_para_ can be reduced by the increase in sheet dimensions and S/D doping level, owing to enhanced current-carrying capacity and improved contact characteristics. Figure 7c analyzes the total *C*_para_ modulation with the effects of spacer materials and *L*_ext_. It indicates that increased *L*_ext_ and lower-*k* spacer materials effectively suppress *C*_para_ by weakening the capacitive coupling between conductive plates. Considering a dual-*k* spacer architecture combining Si_3_N_4_ (low-*k*) and HfO_2_ (high-*k*) with thickness fraction *α* allocated to Si_3_N_4_, Figure 7d shows its impact on the total *C*_para_ for various NS cross-sectional sizes. The competition between gate leakage suppression and *C*_para_ minimization can be balanced by a proper *α*. Additionally, the expansion of the channel cross-section results in larger *C*_para_ because of reduced fringing field distances between the gate and EXT regions. To verify the accuracy of our parasitic models, independent validation points from recent literature are incorporated as scatter symbols in Figure 7b,c. Specifically, our calculated parasitic resistances show consistent scaling trends with the 3 nm and 1.5 nm benchmarks in [16], while our capacitance results for various dielectric spacers exhibit an extraordinary numerical match with the results reported in [30,31]. This cross-verification confirms that our simulation framework accurately captures the electrostatic coupling and transport physics at the sub-2 nm node.

**Figure 8:**
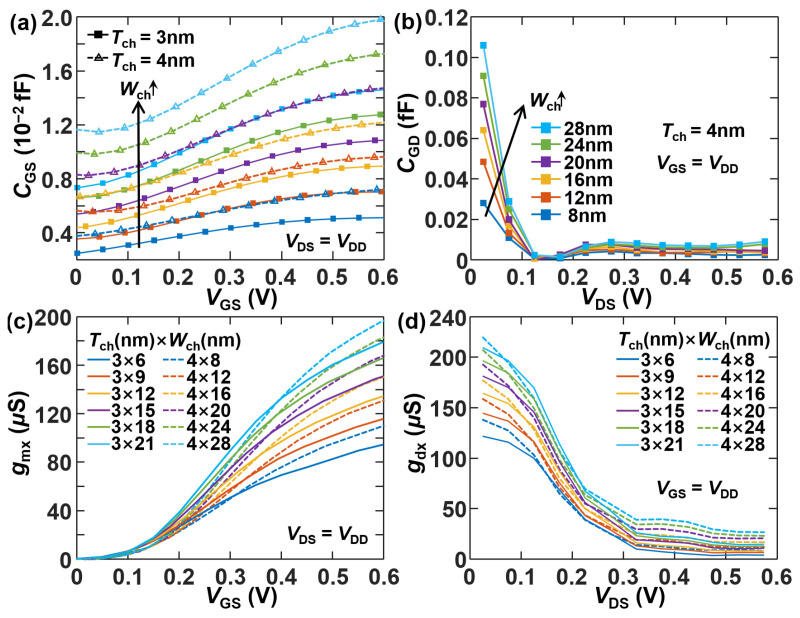
*C-V*, *g*_m_-*V,* and *g*_d_-*V* characteristics of [110]-oriented Si NS nFETs with different channel cross-sectional dimensions *T*_ch_ and *W*_ch_: (**a**) *C*_GS_-*V*_GS_ at *V*_DS_ = *V*_DD_, where *W*_ch_ is 6, 9, 12, 15, 18 and 21 nm from bottom to top for *T*_ch_ = 3 nm, and it is 8, 12, 16, 20, 24 and 28 nm from bottom to top for *T*_ch_ = 4 nm; (**b**) *C*_GD_-*V*_DS_ at *V*_GS_ = *V*_DD_; (**c**) *g*_mx_-*V*_GS_ at *V*_DS_ = *V*_DD_; (**d**) *g*_dx_-*V*_DS_ at *V*_GS_ = *V*_DD_.

Figure 8a and Figure 8b present *C*_GS_-*V*_GS_ and *C*_GD_-*V*_DS_ characteristics at *V*_DS_ = *V*_DD_ and *V*_GS_ = *V*_DD_, respectively, for different cross-sectional sizes of the NS channel. Both *C*_GS_ and *C*_GD_ exhibit approximately direct proportionality to NS width and saturate at high biasing conditions. However, while *C*_GS_ increases sublinearly with increasing *V*_GS_, *C*_GD_ decreases inversely with *V*_DS_, saturating to a minimum value at high *V*_DS_. Figure 8c and Figure 8d show the similar trends of *g*_mx_-*V*_GS_ and *g*_dx_-*V*_DS_ to those of *C*_GS_-*V*_GS_ and *C*_GD_-*V*_DS_, respectively, for various cross-sectional areas of the NS channel. A higher voltage gain (*g*_m_/*g*_d_) is achieved by a smaller channel cross-section, exceeding 30 at *T*_ch_ = 3 nm and *W*_ch_ = 6 nm. This results from the improvement of gate electrostatics and suppression of the short-channel effect.

**Figure 9:**
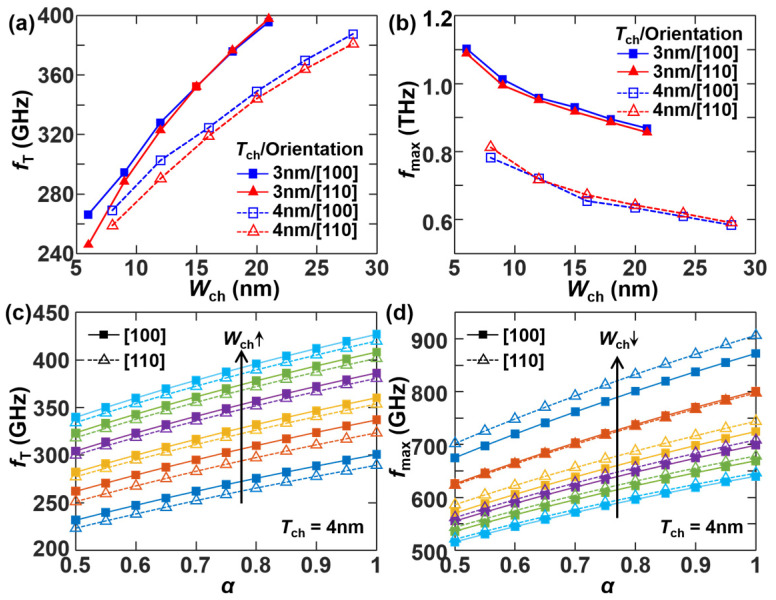
(**a**) *f*_T_ and (**b**) *f*_max_ versus *W*_ch_ for different combinations of *T*_ch_ and channel orientation at *α* = 0.75. (**c**) *f*_T_ and (**d**) *f*_max_ varying with *α* at different *W*_ch_ for [100] and [110] channel orientations, respectively, where *T*_ch_ is fixed at 4 nm; *W*_ch_ is 8, 12, 16, 20, 24, and 28 nm from bottom to top in (**c**), while from top to bottom in (**d**).

Figure 9a,b demonstrates the opposite trends of *f*_T_ and *f*_max_ varying with *W*_ch_ in a sub-2 nm node Si NS LGAA-nFET. *f*_T_ increases by ~40% as *W*_ch_ widens, driven by the enhancement in transconductance. Conversely, *f*_max_ decreases by ~35% over the same *W*_ch_ range due to increased parasitic resistance in narrow channels. A thinner NS channel (*T*_ch_ = 3 nm) is beneficial for improving operation frequency owing to enhanced gate control. The channel crystal orientation [100] slightly outperforms [110], achieving overall larger *f*_T_ and *f*_max_, and this is attributed to the lower DOS and thereby weaker scattering in the electron transport. Figure 9c,d further demonstrate that both *f*_T_ and *f*_max_ are promoted by the increase of *α*, and [110] channel orientation helps improve *f*_max_ for thicker NS channels (*T*_ch_ = 4 nm). In general, Figure 9 reveals the potential applications of sub-2 nm Si NS LGAA-nFETs at millimeter-wave and terahertz frequencies.

It is worth noting that while the extracted *f_T_* and *f_max_* reach the THz range, the use of the quasi-static approximation (i.e., solving the Poisson equation) remains physically justified. This is because the characteristic dimensions of the sub-2 nm LGAA-FET (~100 nm) are significantly smaller than the electromagnetic wavelength at 400 GHz (ranging from 750 μm in vacuum to hundreds of micrometers in dielectric media). In such a deep sub-wavelength regime, retardation and distributed effects across the individual device are negligible, ensuring the reliability of the self-consistent NEGF-Poisson framework.

## 4. Conclusions

In summary, this study provides an integrated parasitics-aware quantum transport framework to evaluate the RF performance of sub-2 nm stacked Si NS LGAA-nFETs. By combining a two-band *k·p* model and dissipative NEGF simulations with a detailed parasitic network analysis, this work characterizes the coupled effects of channel geometry, crystal orientation, and dual-k spacers on key RF figures-of-merit. The results identify a critical design trade-off where scaling *W_ch_* and *T_ch_* enhances voltage gain and fmax, whereas a wider channel is required to optimize f_T_. Furthermore, [100] crystal orientation is shown to mitigate electron–phonon scattering, thereby improving *f_max_* compared to [110] configurations. Our analysis suggests that the implementation of dual-*k* spacers, combined with minimized Schottky barriers and optimized S/D doping, significantly reduces parasitic components, allowing projected *f_T_* and *f_max_* to approach the terahertz regime. Based on our simulation framework that bridges quantum effects and circuit-level performance, the findings of this work offer meaningful design insights for the development of ultra-scaled RF CMOS technology and highlight the necessity of co-designing quantum transport physics with parasitic models for the potential millimeter-wave applications of stacked-LGAA transistor structures.

## Figures and Tables

**Table 1:** Design parameters of stacked NS GAAFETs.

Symbol	Physical Meaning (Unit)	Value
*L*_con_	Metal contact length (nm)	8
*W*_con_	Metal contact width (nm)	20
*L*_sd_	Length of S/D epitaxy (nm)	14
*W*_sd_	Width of S/D epitaxy (nm)	36
*L*_ext_	S/D extension length (nm)	8
*L*_g_	Gate/channel length (nm)	10
*W*_ch_	NS channel width (nm)	6–28
*T*_ch_	NS channel thickness (nm)	3–4
*T*_ox_	Oxide thickness (nm)	1
*T*_sp_	Inner spacer thickness (nm)	10
*N*_stack_	No. of NS stacks	3
*N*_SD_	S/D doping concentration (cm^−3^)	2 × 10^20^

**Table 2:** Stacked nanosheet LGAA-FET device parameters.

Symbol	Physical Meaning	Value (Unit)
*h*	Planck constant	6.626 × 10^−34^ (J·s)
*ℏ*	Reduced Planck constant	1.055 × 10^−34^ (J·s)
*k_B_*	Boltzmann constant	1.380 × 10^−23^ (J/K)
*m*_0_	Electron rest mass	9.109 × 10^−31^ (kg)
*q*_0_	Elementary charge	1.602 × 10^−19^ (C)
*ε_0_*	Vacuum permittivity	8.854 × 10^−12^ (F/m)
*ε_s_*	Silicon relative permittivity	11.9
*χ_s_*	Silicon electron affinity	4.05 (eV)
*ε_ox_*	Gate oxide relative permittivity	3.9
*χ_ox_*	Gate oxide affinity	3.34 (eV)
*ρ*	Silicon mass density	2329 (kg/m^3^)
*a*_0_	Silicon lattice constant	5.43 (Å)
*E_g_*	Silicon band gap	1.12 (eV)
m*	Silicon overall effective mass	0.42 (m_0_)
*m_l_*	Longitudinal effective mass	0.91 (m_0_)
*m_t_*	Transverse effective mass	0.19 (m_0_)
*Ξ_d_*	Dilatational deformation potential	1.1 (eV)
*Ξ_u_*	Uniaxial deformation potential	9 (eV)
*Ξ_u’_*	Shear deformation potential	7 (eV)
*d_op_*	Optical deformation potential	8.0 (eV/Å)
*v*	Sound velocity	9.04 × 10^3^ (m/s)
*C*_11_	Elastic stiffness constants	1.66 × 10^11^ (Pa)
*C*_12_		6.40 × 10^10^ (Pa)
*C*_44_		7.96 × 10^10^ (Pa)
*ℏω*	Optical phonon energy	63 (meV)
*T*	Operating temperature	300 (K)

**Table 3:** Procedures of the NEGF simulation.

**Step 1:**	Initially, set bias voltages (*V_GS_*, *V_DS_*) and initialize the electrostatic potential *φ*^1^. Compute the strain tensor and construct the Hamiltonian **H_3D_** using the 2-band *k⋅p* model.
**Step 2:**	Construct the scattering matrices (***M****_ac_*, ***M****_op_*) for acoustic and optical phonon interactions based on deformation potentials.
**Step 3:**	Solve the ballistic NEGF-Poisson equations self-consistently to obtain a stable initial potential profile *φ_bal_* and drain current *I_bal_*.
**Step 4:**	Extract the cross-sectional potential from the simulation domain and construct the mode-space basis using the Lanczos or eigenvalue method to generate the reduced-order Hamiltonian *h*.
**Step 5:**	Calculate the lead self-energies in the mode space ($\sigma_{S/D}^{R}$, $\sigma_{S/D}^{<}$) to satisfy the open boundary conditions for the current energy grid.
**Step 6:**	Compute the phonon scattering self-energies ($\sigma_{ac}^{R}$, $\sigma_{ac}^{<}$, $\sigma_{op}^{R}$, $\sigma_{op}^{<}$) using the current Green’s functions (${\tilde{G}}^{R,1}$, ${\tilde{G}}^{<,\;1}$) and solve the Dyson and Keldysh equations to update the full Green’s functions.
**Step 7:**	Calculate the electron density $\tilde{n}$ and current $\tilde{I}$. If ($\frac{\Delta \tilde{n}}{\tilde{n}}$) < 0.1% and current continuity error < 0.5%, go to **Step 8**, else update ${\tilde{G}}^{R,l}={\tilde{G}}^{R,l-1}$, ${\tilde{G}}^{<,l}={\tilde{G}}^{<,l-1}$ and go to **Step 6**. (inner self-consistent Born loop)
**Step 8:**	Solve the non-linear Poisson equation using the Gummel scheme to update the electrostatic potential *φ^n^* for the entire device domain.
**Step 9:**	If $\max(\left|\varphi^{n}-\varphi^{n-1}\right|)<\;0.001\;V$, end (simulation converged for the current bias point); Else $\varphi^{n}=\varphi^{n-1}$, and go to **Step 4.** (outer NEGF-Poisson loop)

## Data Availability

The data presented in this study can be obtained from the corresponding authors upon reasonable request due to privacy reasons.
